# Recurrent Cough in the Elderly: A Forgotten Entity

**DOI:** 10.1007/s00408-023-00654-2

**Published:** 2023-11-14

**Authors:** Johanna Tuulikki Kaulamo, Anne Marika Lätti, Heikki Olavi Koskela

**Affiliations:** 1 School of Medicine, Institute of Clinical Sciences, Faculty of Health Sciences, University of Eastern Finland, Yliopistonranta 1, 70210 Kuopio, Finland; 2 Unit for Medicine and Clinical Research, Pulmonary Division, Kuopio University Hospital, PL 100, 70029 Kuopio, Finland; 3 Mehiläinen Terveyspalvelut Oy, Healthcare Services for Prisoners, Kauppakatu 39A, 70100 Kuopio, Finland

**Keywords:** Chronic cough, Epidemiology, Quality of life, Recurrent cough, Risk factors

## Abstract

**Introduction:**

Recurrent cough is little researched in adults. We investigated the prevalence, risk factors, and consequences of recurrent cough, and compared the results to those of isolated chronic cough.

**Methods:**

Cross-sectional email survey in an elderly community-based population. Recurrent cough was defined as ≥ 3 cough episodes within one year (each lasting ≥ 1 week) and no current chronic cough. Isolated chronic cough was defined as current cough lasting ≥ 8 weeks and no recurrent cough.

**Results:**

The prevalence of recurrent cough was 3.8% among all respondents (*n* = 5983). Recurrent cough was associated with asthma (aOR 3.32 (95% CI 2.13–5.18)), chronic rhinosinusitis (2.91 (1.89–4.46)), family history of chronic cough (2.59 (1.88–3.56)), analgesic intolerance (2.13 (1.27–3.57)), male gender (1.92 (1.39–2.66)), gastro-esophageal reflux disease (1.73 (1.21–2.47)), obstructive sleep apnoea (1.69 (1.23–2.32)), symptom sum (1.12 per symptom (1.03–1.22)), and younger age (0.96 per year (0.93–1.00)).

Isolated chronic cough was associated with chronic rhinosinusitis (3.45 (2.39–4.97)), asthma (2.17 (1.38–3.41), gastro-esophageal reflux disease (1.80 (1.32–2.47)), family history of chronic cough (1.80 (1.35–2.41)), obstructive sleep apnoea (1.49 (1.12–2.00)), symptom sum (1.18 per symptom (1.10–1.27)), and body mass index (0.96 per unit (0.93–1.00)).

Among subjects with recurrent and isolated chronic cough, the prevalence of depressive symptoms were 7.7% and 4.2%, *p* = 0.11, the Leicester Cough Questionnaire total scores 15.2 (14.6–15.8) and 16.3 (16.0–16.6), *P* = 0.001, and the mean number of yearly cough-related doctor`s visits 0.58 (0.45–0.71) and 0.36 (0.19–0.53), *P* = 0.007, respectively.

**Conclusion:**

The risk factors and consequences of recurrent and isolated chronic cough were comparable. Recurrent cough seems beneficial to address in cough evaluation.

**Supplementary Information:**

The online version contains supplementary material available at 10.1007/s00408-023-00654-2.

## Introduction

Cough is a protective reflex to clear the airways, but sometimes also a distressing symptom with a high socioeconomic impact. Cough is often due to airborne irritants, transient respiratory infections, or chronic airway diseases. International guidelines encourage diagnostic evaluation of cough primarily according to the length of the current episode [1–5]. They focus mostly on the management of chronic cough (≥ 8 weeks` duration).

However, there are subjects whose cough episodes do not exceed 8 weeks` duration but occur repeatedly. When excluding studies about athletes [6] and children [7–11], little is known about recurrent cough. In a previous study that primarily examined the association of breastfeeding in infancy and respiratory symptoms in adulthood, young adults with recurrent cough reported more smoking, asthma, rhinitis, and heartburn than those without recurrent cough [12]. Other studies have also found the association of recurrent cough with asthma [13, 14]. Our previous study in general adult population showed that wheezing, family history of chronic cough, and cough prolongation to 3–8 weeks at baseline were the main predictors of intermittent cough at 12 months [15]. Consequentially to the paucity of data in adults or elderly, there is no established definition for recurrent cough, and the guidelines do not address how it should be managed. The aims of this study were to define the prevalence of recurrent cough, and to compare the risk factors and consequences of recurrent cough to those of isolated chronic cough in elderly subjects.

## Materials and Methods

### Population

This cross-sectional email survey was conducted to investigate the characteristics of cough in elderly subjects. The epidemiology of cough subtypes in this community-based population have been presented in an earlier publication [16]. The members of the Finnish Pensioners` Federation (26 205 members with an email address, mean age 72.7 years, 63.5% female) were sent an information letter, invitation, and the questionnaire in April 2021. A reminder message was sent 2 weeks later. The data was collected electronically in May 2021. A filled questionnaire was considered as an informed consent. The study was approved by the Ethics Committee of Kuopio University Hospital (289/2015). The Finnish Pensioners` Federation permitted the conduct of this study.

### Questionnaire

All subjects answered 62 questions about age, socioeconomic status, smoking, lifestyle, recently experienced symptoms, general health, disorders diagnosed by a doctor, medication, number of cough episodes, and healthcare use within the past year. Asthma [17], chronic rhinosinusitis (CRS) [18], gastro-esophageal reflux disease (GERD) [19], and obstructive sleep apnoea (OSA) [20, 21] were inquired by questions recommended for epidemiological studies. Depressive symptoms were asked using the Patient Health Questionnaire-2 [22]. The subjects with current cough answered 24 additional questions, which included details about cough frequency and duration, and the Leicester Cough Questionnaire (LCQ) to investigate the cough-related quality of life.

### Definitions

Current cough was defined as presence of cough within 2 weeks. Recurrent cough was defined as ≥ 3 cough episodes within the past year with each episode lasting for ≥ 1 week, and absence of current chronic cough. Subjects with recurrent cough may or may not had current cough at the time of the survey. Isolated chronic cough was defined as current cough lasting ≥ 8 weeks and absence of recurrent cough within the past year. No cough was defined as absence of any cough within the past year. The respondents who did not fulfil the criteria for no cough, isolated chronic cough, or recurrent cough, were excluded. The two cough groups were mutually exclusive.

Current asthma was defined as doctor`s diagnosis of asthma at any age and wheezing within the past year [17]. CRS was present if there was either nasal blockage or discharge (anterior or posterior nasal drip), and either reduction/loss of smell or facial pain/pressure for ≥ 3 months within the past year [18]. GERD was defined as presence of heartburn or regurgitation at least once a week in the past 3 months [19]. OSA was defined as presence of ≥ 2 of the following features: Loud snoring, daytime tiredness, observed apnoeas, and arterial hypertension (the STOP-questionnaire) [20, 21]. Symptom sum was defined as the sum (0–15) of experienced non-respiratory symptoms during the past month (Supplementary file). Disorder sum (0–19) was defined as the number of medical conditions diagnosed by a doctor, excluding background disorders of chronic cough (Supplementary file). Depressive symptoms were present if the Patient Health Questionnaire-2 score was ≥ 3 [22]. Family history of chronic cough was defined as cough lasting > 8 weeks in parents or siblings. Trigger sum (0–15) was defined as the sum of external cough-triggering factors. Allergy was defined as self-reported allergy to animals, pollens, or food. Upper respiratory tract infection (URTI) was present if the subject reported fever, sore throat, nasal congestion, rhinorrea, muscle/joint pain or headache at onset of current cough. Analgesic intolerance was present if the subject reported rash, facial swelling, or dyspnoea due to any pain medication use. Chronic sputum production was defined as phlegm production on most days or nights for ≥ 3 months of the year [23]. Previous COVID-19 infection was defined as self-reported, laboratory-confirmed diagnosis of the infection. Any other diagnosis mentioned was defined as self-reported doctor`s diagnosis of the disease.

### Statistical Analysis

Descriptive data are shown as means and 95% confidence intervals. The cough bout frequency and LCQ scores could be defined only in those subjects with recurrent cough, who suffered from current cough. Chi-squared test and Mann–Whitney U-test were used for bivariate comparisons. In the multivariate models, the risk factors for recurrent and isolated chronic cough were analysed by utilising subjects with no cough as the reference group. The independent variables were chosen in the multivariate analyses based on biologically plausible association with cough, statistically significant association with the outcome variable in the bivariate analysis, and prevalence of ≥ 2% in the study population. Age, gender, and body mass index were included in the multivariate analyses as possible confounders. There was a strong interrelationship between “symptom sum” and “disorder sum”. Of them, only “symptom sum” was included in the multivariate analyses due to its stronger association with recurrent cough and isolated chronic cough. The multivariate analyses were conducted using binary logistic regression with a backward directed stepwise process. A *p*-value < 0.05 was considered statistically significant, but suggestive associations (*P* < 0.1) are also presented. SPSS version 27 was utilised for the analyses.

## Results

**Fig. 1:**
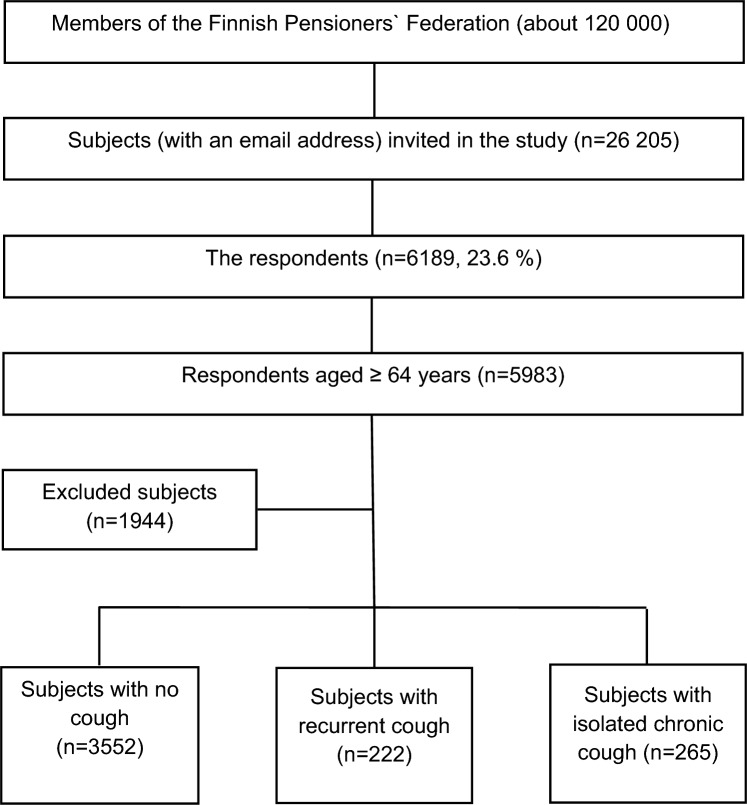
Flow chart

The response rate was 23.6% (*n* = 6189, mean age 72.2 (5.5) years, 66.4% female) (Fig. 1). The proportion of missing values was < 2.5%, except for the questions about family income (2.9%) and OSA (3.1–3.7%). Among the 5983 subjects aged ≥ 64 years, there were 3552 subjects with no cough, 222 subjects with recurrent cough (90 of them having current cough), and 265 subjects with isolated current chronic cough (Table 1). The prevalence of recurrent cough and isolated chronic cough among all respondents were 3.8% and 4.6%, respectively.

**Table 1:** Characteristics of subjects without any cough, subjects with recurrent cough and subjects with isolated chronic cough

Characteristic	No cough (*n* = 3552)	Recurrent cough (*n* = 222)	Isolated chronic cough (*n* = 265)
Age, years	72.55 (72.38–72.71)	71.93 (71.31–72.55)	72.82 (72.16–73.47)
Female gender, %	67.2	58.6**	71.3
Body mass index, kg/m^2^	27.08 (26.94–27.23)	28.02 (27.40–28.64)**	27.26 (26.74–27.77)
Current smoking, %	1.5	1.8	1.9
Ever-smoking, %	33.6	41.9*	36.6
Symptom sum	1.73 (1.68–1.79)	2.60 (2.32–2.88)***	2.63 (2.41–2.85)***
Disorder sum	1.35 (1.30–1.39)	1.89 (1.69–2.08)***	1.69 (1.53–1.85)***
Family history of chronic cough, %	18.3	42.3***	34.7***
Allergy, %	6.6	14.4***	14.3***
Current asthma, %	3.8	18.0***	13.3***
Wheezing, %	12.9	48.2***	41.3***
Chronic rhinosinusitis, %	5.0	19.4***	20.4***
Chronic obstructive pulmonary disease, %	0.8	2.3*	1.1
Bronchiectasis, %	0.3	1.4*	1.5**
Pulmonary fibrosis, %	0.3	0.9	0.4
Sarcoidosis, %	0.4	0.9	0.8
Gastro-esophageal reflux disease, %	12.8	27.7***	27.1***
Obstructive sleep apnoea, %	25.3	49.8***	39.5***
Chronic sputum production, %	6.9	46.4***	39.4***
COVID-19 infection, %	0.2	1.8***	1.2**
Analgesic intolerance, %	4.2	11.9***	7.7**
Depressive symptoms, %	3.6	7.7**	4.2

Subjects with recurrent cough and isolated chronic cough are compared to those with no cough. The figures are presented as percentages or means and 95% confidence intervals

*P < 0.05, **P < 0.01, ***P < 0.001

**Fig. 2:**
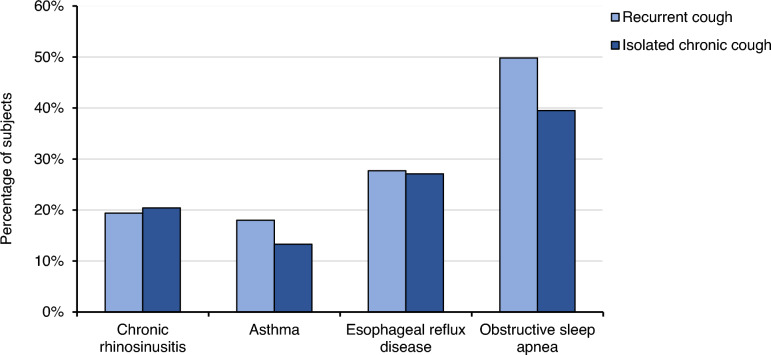
Percentages of cough background disorders among subjects with recurrent cough (*n* = 222) and isolated chronic cough (*n* = 265)

**Fig. 3:**
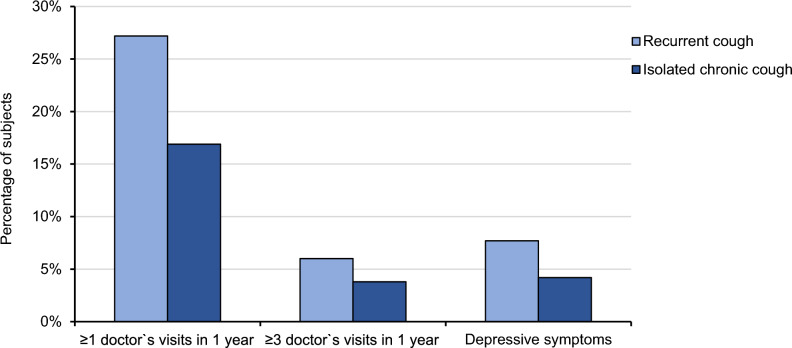
Percentages of cough-related doctor`s visits and depressive symptoms among subjects with recurrent cough (*n* = 222) and isolated chronic cough (*n* = 265)

The characteristics of subjects with recurrent and isolated chronic cough resembled each other in many respects (Table 2, Fig. 2). However, the proportion of males was higher in recurrent cough. The LCQ total and domain scores were lower in subjects with recurrent cough than in those with isolated chronic cough (Table 2). There were more cough-related doctor`s visits during the past year among subjects with recurrent cough than among subjects with isolated chronic cough (Table 2, Fig. 3).

**Table 2:** Comparisons between recurrent cough and isolated chronic cough

Characteristic	Recurrent cough (*n* = 222)	Isolated chronic cough (*n* = 265)	*p*-value
Age, years	71.93 (71.31–72.55)	72.82 (72.16–73.47)	0.135
Female gender, %	58.6	71.3	**0.003**
Body mass index, kg/m^2^	28.02 (27.40–28.64)	27.26 (26.74–27.77)	0.095
Current smoking, %	1.8	1.9	0.956
Ever-smoking, %	41.9	36.6	0.233
Trigger sum	3.7 (3.3–4.1)	3.3 (2.94–3.67)	0.212
Family history of chronic cough, %	42.3	34.7	0.085
Allergy, %	14.4	14.3	0.981
Chronic obstructive pulmonary disease, %	2.3	1.1	0.333
Bronchiectasis, %	1.4	1.5	0.884
Current asthma, %	18.0	13.3	0.148
Chronic rhinosinusitis, %	19.4	20.4	0.781
Gastro-esophageal reflux disease, %	27.7	27.1	0.878
Obstructive sleep apnoea, %	49.8	39.5	**0.026**
Chronic sputum production, %	46.4	39.4	0.123
Analgesic intolerance, %	11.9	7.7	0.122
Symptom sum	2.60 (2.32–2.88)	2.63 (2.41–2.85)	0.409
Disorder sum	1.89 (1.69–2.08)	1.69 (1.53–1.85)	0.187
Depressive symptoms, %	7.7	4.2	0.106
Mean number of cough-related doctor`s visits during the past year	0.58 (0.45–0.71)	0.36 (0.19–0.53)	**0.007**
Subjects with ≥ 3 cough-related doctor`s visits during the past year, %	6.0	3.8	0.272
Symptoms of acute upper respiratory infection at onset of current cough, %	33.7^a^	10.7	** < 0.001**
Cough bout frequency (median, range)	4–6 times per week (several times per day to ≤ 1 time per week)^a^	≥ 1 time per day (several times per day to ≤ 1 time per week)	0.847
LCQ total score	15.20 (14.64–15.76)^a^	16.27 (15.98–16.55)	**0.001**
LCQ, physical score	5.00 (4.83–5.18)^a^	5.37 (5.29–5.46)	** < 0.001**
LCQ, psychological score	4.95 (4.76–5.15)^a^	5.19 (5.07–5.30)	**0.032**
LCQ, social score	5.24 (5.01–5.48)^a^	5.70 (5.59–5.81)	** < 0.001**

*p* < 0.05 are highlighted in bold

The figures are presented as percentages or means and 95% confidence intervals unless stated otherwise

^a^ among subjects with current cough (*n* = 90)

In the multivariate analyses, most risk factors were common for recurrent and isolated chronic cough (Tables 3 and 4). However, male gender, younger age, and analgesic intolerance were specific risk factors of recurrent cough.

**Table 3:** Risk factors of recurrent cough in the multivariate analysis (*n* = 222), compared to subjects with no cough (*n* = 3552)

Characteristic	Adjusted odds ratio and 95% confidence interval
Current asthma	3.32 (2.13–5.18) ***
Chronic rhinosinusitis	2.91 (1.89–4.46) ***
Family history of chronic cough	2.59 (1.88–3.56) ***
Analgesic intolerance	2.13 (1.27–3.57) **
Male gender	1.92 (1.39–2.66) ***
Gastro-esophageal reflux disease	1.73 (1.21–2.47) **
Obstructive sleep apnoea	1.69 (1.23–2.32) **
Symptom sum	1.12 per symptom (1.03–1.22) **
Age	0.96 per year (0.93–1.00) *

^*^P < 0.05, **P < 0.01, ***P < 0.001

**Table 4:** Risk factors of isolated chronic cough in the multivariate analysis (*n* = 265), compared to subjects with no cough (*n* = 3552)

Characteristic	Adjusted odds ratio and 95% confidence interval
Chronic rhinosinusitis	3.45 (2.39–4.97) ***
Current asthma	2.17 (1.38–3.41) **
Gastro-esophageal reflux disease	1.80 (1.32–2.47) ***
Family history of chronic cough	1.80 (1.35–2.41) ***
Obstructive sleep apnoea	1.49 (1.12–2.00) **
Symptom sum	1.18 per symptom (1.10–1.27) ***
Body mass index	0.96 per unit (0.93–1.00) *

*P < 0.05, **P < 0.01, ***P < 0.001

## Discussion

The first main finding of this study was that the risk factors of recurrent cough and isolated chronic cough were essentially the same. The second main finding was that the consequences of recurrent cough, such as depressive symptoms, quality of life, and healthcare use, were comparable to those of isolated chronic cough.

Because recurrent cough lacks an established definition in adults, it was defined by the authors considering the frequency and duration of common cold-associated cough. Adults catch approximately 2–3 colds annually [24]. Thus, recurrent cough was defined as ≥ 3 cough episodes during the past year. The definition of ≥ 1 week for episode duration was chosen because common cold-associated cough usually resolves in 1 week [24], although transient increase in cough reflex sensitivity may last up to 4 weeks [25]. We hypothesised that ≥ 3 cough episodes of more than 1-weeks` duration within a year indicates abnormality, irrespective of the presence of an URTI.

The classic triad of chronic cough background disorders, CRS, asthma, and GERD, was associated with both recurrent cough and isolated chronic cough. Episodic cough in CRS and asthma is understandable given their vulnerability to exacerbations due to infections or environmental factors, whereas GERD has previously been associated mostly with prolonged or chronic, continuous cough [2, 15]. The episodic nature of reflux-cough could be attributable to changes in diet and lifestyle since the benefits of anti-acid drugs alone are limited. Because all background disorders of isolated chronic cough were also risk factors for recurrent cough, fluctuating control of the background disorders seems like a logical explanation for the relapsing and remitting course of cough. Cold temperature could also contribute to seasonal changes in cough among subjects with increased cough reflex sensitivity [26]. The results also suggest that the typical background disorders of chronic cough may exist in recurrent episodes of only 1 weeks` length, however the episode durations could not be specified in this study. Considering that most people do not seek medical advice for current cough of any length [27, 28], and that healthcare resourcing may limit early access to doctor`s evaluation, a routine question about the history of recurrent cough could help to reach the undiagnosed background disorders.

Despite the noticeable similarity of the risk factors in recurrent and isolated chronic cough, there were some specific risk factors for recurrent cough in this elderly population. Surprisingly, those were male gender, younger age, and analgesic intolerance. The cough reflex is reported to be more sensitive in women than in men [29], and the patients in cough clinics are predominantly females [30]. In this context, the reason for the association of male gender with recurrent cough is unclear. Smoking does not seem to explain it, because very few subjects were current smokers, and the association was independent from ever-smoking in the multivariate analysis. The protective association of age with recurrent cough could at least partly be attributable to decreasing URTI-incidence towards the higher age, as previously reported [31]. Intolerance to analgesics was also associated with recurrent cough, for an unknown reason. Overall, these are atypical risk factors of cough and warrant future research.

Chronic cough can predispose to impairment in the quality of life [27], depression [32, 33], and repeated healthcare use [28, 34]. All quality-of-life scores by LCQ were statistically significantly lower in subjects with recurrent cough than in those with isolated chronic cough. However, LCQ total scores did not reach the clinically meaningful difference of 1.3 points [35, 36]. Depressive symptoms were more common among subjects with recurrent cough, although not statistically significantly. Furthermore, recurrent cough led to more doctor`s visits than isolated chronic cough. In this elderly population, the personal and socioeconomic impact of recurrent cough was at least as substantial as of isolated chronic cough.

The prevalence of recurrent cough was 3.8%. This was in the circumstances of the early pandemic, when the incidence of COVID-19 infections in this population [16], and URTIs in Finland [37], were low. Considering this, URTI-associated cough episodes were likely fewer than in the current time. However, no comparative data could be found on the prevalence of recurrent cough in community-based adult populations. From another perspective, recurrent cough did not disappear during low URTI-incidence, which may suggest other reasons for cough recurrence. Among subjects with recurrent and current cough, 34% reported that the current episode was triggered by an URTI. This suggests that most current acute or subacute episodes were triggered by something else. However, the current episode was expectedly more often URTI-related in subjects with recurrent cough than in those with isolated chronic cough. Of note, the prevalence of isolated chronic cough was 4.6%. Because of the mutually exclusive definitions, isolated chronic cough included only continuous chronic coughers who did not report overlapping recurrent cough. In contrast, according to the currently recommended definition of ≥ 8 weeks, the total prevalence of chronic cough was much higher among all respondents of this survey (13.5%) [16]. This means that most cases of chronic cough involved either precursing recurrent cough or episodic exacerbations of chronic cough.

There were several shortcomings in this study. Despite of the rather low response rate, the age and gender distributions of the target population and the respondents were highly comparable. However, the subjects with particularly problematic cough may have been more willing to participate than others. The population consisted of elderly persons who were able to respond to an email survey. Therefore, younger and disabled persons were excluded, and the generalisability of the results should be studied in other populations. However, studying cough specifically in the elderly can be regarded also as a strength, considering the globally ageing populations. Important background disorders of cough, namely bronchiectasis and chronic obstructive pulmonary disease, could not be included in the final risk factor analyses due to low prevalence in the study population. The STOP-questionnaire may overestimate the prevalence of OSA [21]. Current smokers and thus the impact of smoking was underrepresented in the results. Angiotensin-converting-enzyme inhibitor use was not investigated separately from other antihypertensive drugs and was not included in the analyses. Recall bias may somewhat affect the reporting of recurrent cough episodes. The study design does not allow confirmation of causality. Also, all data was self-reported and hence include variation in how the subjects experience and report symptoms [38]. However, this was controlled with the variable “symptom sum”. A strength of the study is that it was conducted with a comprehensive questionnaire which was designed to study specifically the epidemiology and consequences of cough. It included validated questionnaires to investigate cough-related quality of life and several cough background disorders, which may help to include symptomatic background disorders that are yet undiagnosed. This study was the first one to define risk factors and consequences for recurrent cough in adults, a phenomenon that is recognisable in the clinical setting but so far little studied.

## Conclusion

Recurrent cough is easy to recognise by simply asking. It impacts the quality of life and prompts doctor’s visits comparably to isolated chronic cough and may be treatable given the documented background disorders. Thus, history of recurrent cough seems beneficial to consider in cough evaluation.

### Supplementary Information

Below is the link to the electronic supplementary material.

Supplementary file1 (PDF 100 KB)
