# Supplementary material for: Recurrent Cough in the Elderly: A Forgotten Entity
Source: Lung. 2023 Nov 14;201(6):545–53. doi: 10.1007/s00408-023-00654-2 (PMC10673973; doi:10.1007/s00408-023-00654-2)
Supplement: Supplementary file 1 — Supplementary file1 (PDF 100 KB) [file 408_2023_654_MOESM1_ESM.pdf]

Supplementary file. Questions included in the variables “symptom sum” and “disorder sum”.

**Symptom sum (0-15)**	**Disorder sum (0-19)**
Chest pain	Allergy
Arthralgia	Pulmonary fibrosis
Back pain	Sarcoidosis
Tooth ache	Pulmonary tuberculosis
Lower limb swelling	Gastric ulcer
Varicose veins	Parkinson`s disease
Rash	Depression
Headache	Other psychiatric disorder
Insomnia	Rheumatoid arthritis
Depressive mood	Other disease of the connective tissue
Other mental symptom	Hypothyroidism
Constipation	Hypertension
Other gastrointestinal symptoms (gas, diarrhea)	High blood cholesterol
Sciatica	Diabetes
Urinary difficulties	History of myocardial infarction
	Coronary artery disease
	Cancer
	Degenerative or other spine condition
	Prostatic hyperplasia

Journal: LUNG

Article: Recurrent cough in the elderly-a forgotten entity

Authors: Johanna Tuulikki Kaulamo, Anne Marika Lätti, Heikki Olavi Koskela

Corresponding author: Johanna Kaulamo, School of Medicine, Institute of Clinical Sciences, Faculty of Health Sciences, University of Eastern Finland, Yliopistonranta 1, 70210 Kuopio, Finland. Email: kaulamo@uef.fi
